# The analgesic efficacy of transverse abdominis plane block versus epidural analgesia: A systematic review with meta-analysis

**DOI:** 10.1097/MD.0000000000011261

**Published:** 2018-06-29

**Authors:** Moira Baeriswyl, Frank Zeiter, Denis Piubellini, Kyle Robert Kirkham, Eric Albrecht

**Affiliations:** a Department of Anesthesia, Lausanne University Hospital, Lausanne, Switzerland; b Department of Anesthesia, Toronto Western Hospital, University of Toronto, Toronto, Canada.

**Keywords:** abdominal surgery, analgesia, epidural analgesia, postoperative pain

## Abstract

**Background:**

The aim of the study was to compare the analgesic efficacy of epidural analgesia and transverse abdominis plane (TAP) block. TAP block has gained popularity to provide postoperative analgesia after abdominal surgery but its advantage over epidural analgesia is disputed.

**Methods:**

We followed the Preferred Reporting Items for Systematic Reviews and Meta-Analyses (PRISMA) statement guidelines. Only trials comparing TAP block with epidural analgesia were included. The primary outcome was pain score at rest (analog scale, 0–10) on postoperative day 1 analyzed in subgroups according to the population (children and adults). Secondary outcomes included rate of hypotension, length of stay, and functional outcomes (time to first bowel sound, time to first flatus).

**Results:**

Ten controlled trials, including 505 patients (195 children and 310 adults), were identified. Pain scores at rest on postoperative day 1 were equivalent for TAP block and epidural analgesia groups in children (mean difference: 0.3; 95% confidence interval [CI]: −0.1 to 0.6; *I*^2^ = 0%; *P* = .15) and in adults (mean difference: 0.5; 95% CI: −0.1 to 1.0; *I*^2^ = 81%; *P* = .10). The quality of evidence for our primary outcome was moderate according to the GRADE system. The epidural analgesia group experienced a higher rate of hypotension (relative risk: 0.13; 95% CI: 0.04–0.38; *I*^2^ = 0%; *P* = .0002), while hospital length of stay was shorter in the TAP block group (mean difference: −0.6 days; 95% CI: −0.9 to −0.3 days; *I*^2^ = 0%; *P* < .0001), without impact on functional outcomes.

**Conclusion:**

There is moderate evidence that TAP block and epidural analgesia are equally effective in treating postoperative pain in both pediatric and adult patients, while TAP block is associated with fewer episodes of hypotension and reduced length of stay.

## 1 Introduction

For many years, epidural and caudal analgesia have been considered the gold-standard techniques after abdominal surgery for adults and children, respectively. The techniques consist of injecting the local anesthetic within the epidural space, between the ligamentum flavum and the dura mater. Depending on the surgical site and the level of injection, cervical, thoracic, or lumbar nerve roots are blocked after their emergence from the neural foramen. Epidural and caudal analgesia have technical drawbacks with epidural local anesthetic associated with hypotension secondary to the sympathetic blockade,^[1,2]^ and increased intracranial pressure described after caudal blockade.^[3]^

In the last decade, a new abdominal truncal block, called the tranversus abdominis plane (TAP) block, was described consisting of local anesthetic injection between the internal oblique and transversus abdominis muscle.^[4]^ This block provides analgesia by blocking the 7th to 11th intercostal nerves (T7–T11), the subcostal nerve (T12), and the ilioinguinal nerve and iliohypogastric nerve (L1–L2).^[4]^ Two distinct approaches have been described: an intercostoiliac approach where the probe is positioned between the rib cage and the iliac crest, and an oblique subcostal approach where the probe is placed anterior to the midaxillary line in an oblique subcostal angle. Both approaches have been shown to effectively cover pain after abdominal wall surgery. The TAP block has achieved widespread clinical uptake due to the technique's simplicity when performed with ultrasound guidance and the absence of significant side effects.^[5]^

Several authors have compared the TAP block to neuraxial analgesic techniques but have reported conflicting results.^[6,7]^ To reconcile these conclusions, we undertook this meta-analysis with the objectives to compare analgesic efficacy and side effects of TAP block versus epidural analgesia in pediatric and adult patients.

## 2 Methods

### 2.1 Literature search and inclusion criteria

This investigation followed the recommended process described in the “Preferred Reporting Items for Systematic Reviews and Meta-Analyses” (PRISMA) statement,^[8]^ and the protocol was registered on PROSPERO (registration number: CRD42017067401). Due to the nature of this manuscript (meta-analysis), an ethical approval was not necessary. The authors searched electronic databases including: Medline (until September 2017), PubMed (until September 2017), Excerpta Medica database, Embase (until September 2017), the Cochrane Central Register of Controlled Clinical Trials, CENTRAL (until September 2017), and the Latin American and Caribbean Center on Health Sciences Information, LILACS (until September 2017) applied the following population search terms: Epidural anesthesia OR Epidural anaesthesia OR Caudal anesthesia OR Caudal anaesthesia OR Epidural drug administration OR Epidural injection OR Epidural analgesia OR Peripheral nerve block OR Regional anesthesia OR Regional anaesthesia OR Pain. These search results were combined with Abdominal wall block OR Nerve block OR Transversus Abdominal wall block. Results were further limited with Clinical trials OR Random allocation OR Therapeutic use. The following words were searched as keywords: Analg^∗^, Pain^∗^, Nerve^∗^, Epidural^∗^, Caudal^∗^, Extradural^∗^, Postsurg^∗^, Postoperat^∗^, Perioperat^∗^, Transvers^∗^, Block^∗^. The results of this search strategy were limited to randomized controlled trials and humans. No age or language limits were placed on the search. Finally, the references of all articles retrieved from the search were manually scrutinized for any relevant trials not identified using the strategy described above and Google Scholar was examined for any additional publication.

### 2.2 Population

The meta-analysis addresses female and male adults (18 years or older) and children (younger than 18 years) undergoing any abdominal surgical operation.

### 2.3 Intervention and comparator

Only trials comparing TAP block, using an intercostoiliac (probe placed between the rib cage and iliac crest) or oblique subcostal approach (probe placed anterior to the midaxillary line in an oblique subcostal angle) with epidural or caudal analgesia were included in the present meta-analysis.

### 2.4 Outcomes

The specific outcomes sought from each article were derived following our standard approach, which we described in previous meta-analyses on acute postoperative pain.^[9,10]^ The primary outcome was pain score at rest on postoperative day 1. Secondary acute pain-related outcomes were pain score at rest at 12 hours postoperatively, and on postoperative day 2; pain score on movement at 12 hours postoperatively, and on postoperative days 1 and 2; and intravenous (IV) morphine consumption equivalents at 12 hours postoperatively, and on postoperative days 1 and 2. We also aimed to capture functional-related outcomes such as time to first bowel sounds, time to first flatus, and hospital length of stay. Secondary side-effect-related outcomes were rates of postoperative nausea and vomiting (PONV) within the first 24 hours postoperatively, hypotension, and infection.

### 2.5 Trial characteristics

Extracted trial characteristics included type of surgery, regional block technique, concentration and volume of local anesthetics injected, and type of multimodal analgesia.

### 2.6 Rating of the studies

The quality of the research methodology of each randomized trial was assessed following the Cochrane Collaboration's Risk of Bias Tool for randomized controlled trials.^[11]^ Two authors (FZ and KK) independently screened, reviewed, and scored the items for each trial using this method and a third one (CP) extracted data for the analyses. Disagreements with scoring or extracted data were resolved through discussion with another author (EA).

### 2.7 Data extraction

The source study text, tables, or graphs were used to extract mean values, standard deviations, standard error of means, 95% confidence intervals (CIs), number of events, and total number of participants. The authors of trials who failed to report the sample size or results as a mean and standard deviation or standard error of the mean or 95% CIs were requested twice by mail to give the missing raw data. If no reply was obtained, the median and interquartile range were used for mean and standard deviation approximations, as follows: the mean was estimated as equivalent to the median and the standard deviation was approximated to be the interquartile range divided by 1.35, or the range divided by 4.^[12]^ All opioids were converted into equianalgesic doses of IV morphine for analysis (IV morphine 10 mg = oral morphine 30 mg = IV hydromorphone 1.5 mg = oral hydromorphone 7.5 mg = IV pethidine 75 mg = oral oxycodone 20 mg = IV tramadol 100 mg).^[5]^ Pain scores reported as visual, verbal, or numeric rating scales were converted to a standardized 0 to 10 analog scale for quantitative evaluations. Finally, we rated the quality of evidence for each outcome following the Grades of Recommendation, Assessment, Development, and Evaluation (GRADE) Working Group system.^[13]^

### 2.8 Statistical analysis

Meta-analyses were performed with the assistance of Review Manager software (RevMan version 5.3.5; Copenhagen, The Nordic Cochrane Centre, The Cochrane Collaboration 2014). This software estimates the weighted mean differences for continuous data, weighted standardized mean difference for ordinal data, and risk ratio for categorical data between groups, with an overall estimate of the pooled effect. A meta-analysis was conducted only if 2 or more trials reported the outcome of interest. The coefficient *I*^2^ was used to evaluate heterogeneity with predetermined thresholds for low (25–49%), moderate (50–74%), and high (>75%) levels.^[14]^ A random effects model was applied in cases of moderate or high heterogeneity; otherwise a fixed effects model was used. All pain-related outcomes were analyzed in subgroups according to the type of population (children and adults) and the tap block approach adopted by the authors (intercostoiliac vs oblique subcostal) in an attempt to account for heterogeneity. The likelihood of publication bias was assessed for our primary outcome by drawing a funnel plot of standard error of the mean difference in pain score at rest on postoperative day 1 (y-axis) as a function of the mean difference in pain score at rest on postoperative day 1 (x-axis) and confirmed with Duval and Tweedie's trim and fill test.^[15,16]^ This assessment was performed using Comprehensive Meta-analysis Version 2 software (BioStat, Englewood, NJ). Results are presented as the mean difference or relative risk (RR) with 95% CI. A 2-sided *P-*value of <.05 was considered significant.

## 3 Results

Of the 807 trials identified from the literature search strategy, 10 met the inclusion criteria,^[6,7,17–24]^ representing a total of 505 patients, and including 195 children aged from 1 to 9 years, and 310 adults.

**Figure 1:**
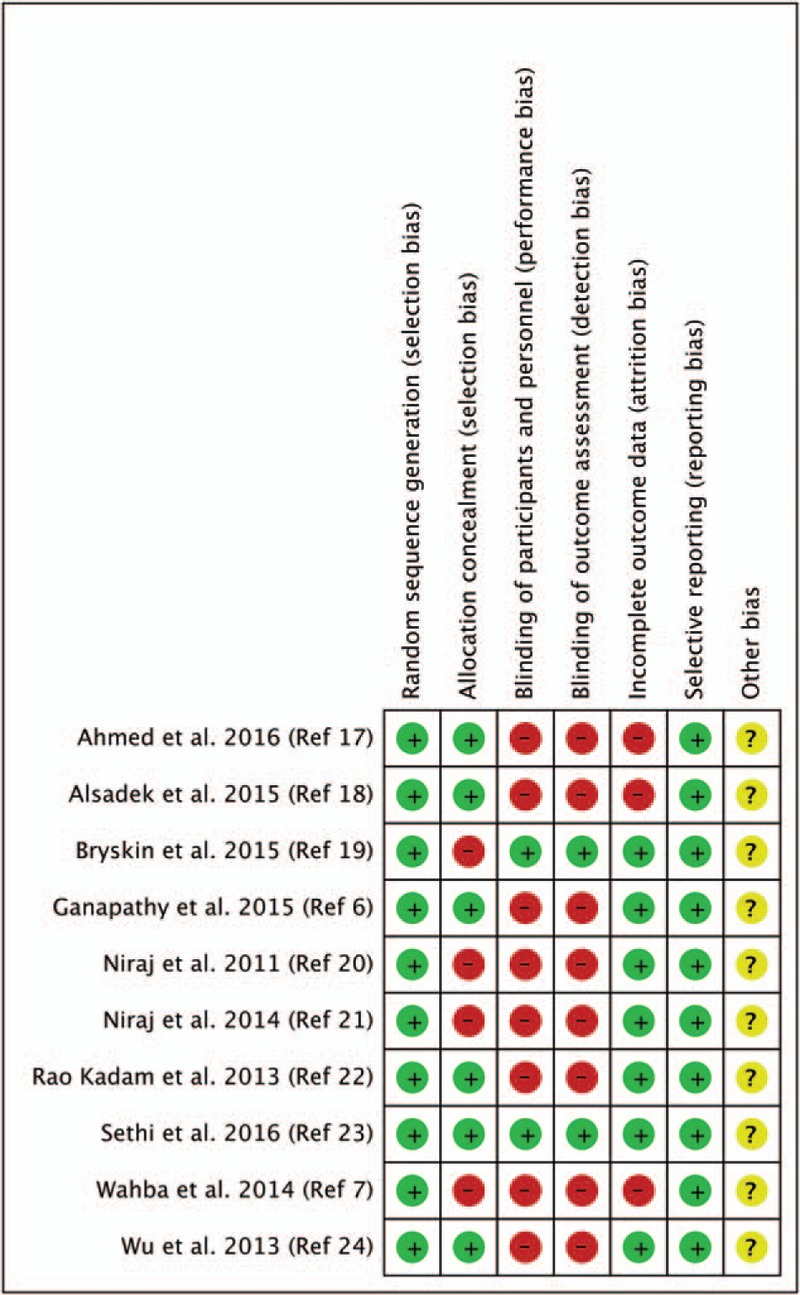
Cochrane collaboration risk of bias summary: evaluation of bias risk items for each included study. Green circle = low risk of bias; red circle = high risk of bias; yellow circle = unclear risk of bias.

According to our assessment following the Cochrane Collaboration Risk of Bias tool (Fig. 1), the majority of trials had a high risk of bias related to blinding of participants and outcome assessors. Attempts were made to contact 9 authors,^[6,7,17–24]^ and 3 provided the additional data requested.^[20,22,24]^ Data were approximated from median and range in 4 trials.^[6,7,21,23]^

Table 1 presents the trial characteristics. All trials performed a bilateral ultrasound-guided TAP block, except 2 that injected the local anesthetic unilaterally.^[17,23]^ All trials with children^[17–19,23]^ and 2 trials with adults^[7,22]^ employed an intercostoiliac approach; 4 trials with adults used an oblique subcostal approach.^[6,20,21,24]^ In 5 trials, authors administered a single-shot injection only,^[17–19,23,24]^ while in 5 others, a continuous infusion was associated with the single-shot injection.^[6,7,20–22]^ Of note, 1 trial compared a single-shot injection for TAP block with a continuous epidural analgesia.^[24]^ Local anesthetics injected were bupivacaine or levobupicaine 0.125% to 0.375%^[6,7,17–21]^ or ropivacaine 0.2% to 0.375%.^[6,22,24]^ Authors consistently combined the regional technique with general anesthesia.

**Table 1:**
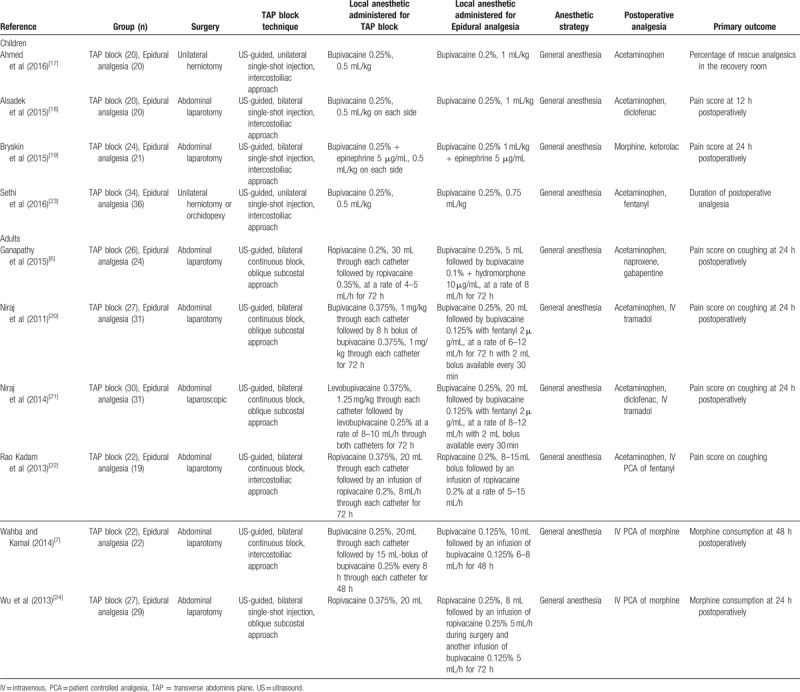
Trial characteristics.

**Figure 2:**
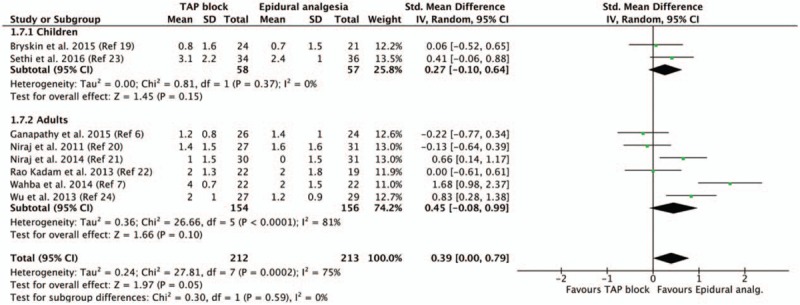
Pain score at rest on postoperative day 1 according to the population (children vs adults). CI = confidence interval, SD = standard deviation, TAP = transverse abdominis plane.

**Figure 3:**
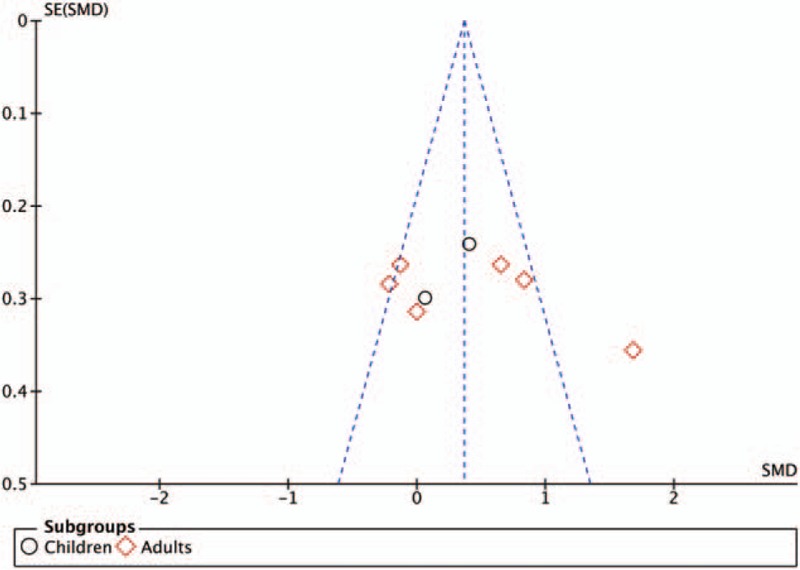
Funnel plot of the primary outcome (pain score at rest on postoperative day 1). SE(SMD) = standard error of the standard mean difference.

Pain scores at rest on postoperative day 1 were equivalent in TAP block and epidural analgesia groups, without subgroup differences between children and adults (Fig. 2). The quality of evidence for our primary outcome was moderate according to the GRADE system. With regards to the funnel plots for our primary outcome (Fig. 3), the Duval and Tweedie's trim and fill test revealed the point estimates for the combined studies to be 0.37 (95% CI: 0.18–0.77), suggesting an absence of publication bias.

Among the secondary pain-related outcomes, only 3 trials conducted in children recorded resting pain scores at 12 hours postoperatively (mean difference: −0.8; 95% CI: −2.4 to 0.8; *I*^2^ = 95%; *P* = .32).^[18,19,23]^ All other secondary pain-related outcomes were reported on adult trials only and are presented in Table 2. Regarding the functional outcomes, time to first bowel sounds measured by 1 trial was equivalent in both groups (mean difference: −14.8 hours; 95% CI: −43.6 to 14.0 hours; *I*^2^ not applicable; *P* = .31),^[6]^ as was time to first flatus measured by 4 trials (mean difference: 5.2 hours; 95% CI: −5.1 to 15.5 hours; *I*^2^ = 77%; *P* = .32).^[6,7,21,24]^ Based on 3 trials,^[7,21,22]^ statistical analysis revealed that hospital length of stay was inferior in the TAP block group (mean difference: −0.6 days; 95% CI: −0.9 to −0.3 days; *I*^2^ = 0%; *P* < .0001).

**Table 2:**
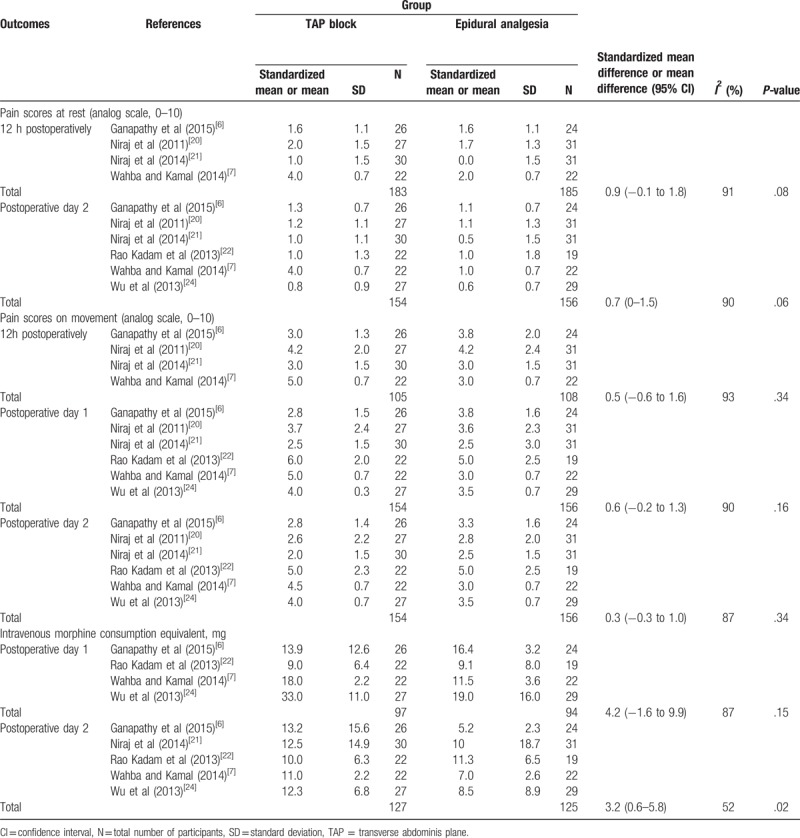
Secondary pain-related outcomes in adult patients.

A subgroup analysis of the TAP block approach for all pain-related outcomes (intercostoiliac vs subcostal approaches) did not account for the observed heterogeneity, nor did it reveal a significant difference between either subgroup compared to epidural analgesia, or between subgroups. For example, subgroup analysis of the primary outcome, pain score at rest on postoperative day 1, showed that the standardized mean difference (95% CI) for intercostoiliac and oblique subcostal approaches were 0.52 (−0.15 to 1.19) and 0.29 (−0.23 to 0.81), respectively, with *P*-values of .13 and .28, and *I*^2^ values of 81% and 74%, when compared to epidural analgesia; the *P-*value for subgroup difference was .60.

The rate of hypotension, recorded by 4 trials,^[6,7,22,24]^ was higher in the epidural analgesia group (RR: 0.13; 95% CI: 0.04–0.38; *I*^2^ = 0%; *P* = .0002), while the rate of PONV was equivalent (RR: 0.80; 95% CI: 0.37–1.74; *I*^2^ = 51%; *P* = .57).^[18,19,21,23,24]^ One trial reported no infection in either group.^[18]^

Table 3 summarized the findings according to the GRADE system.

**Table 3:**
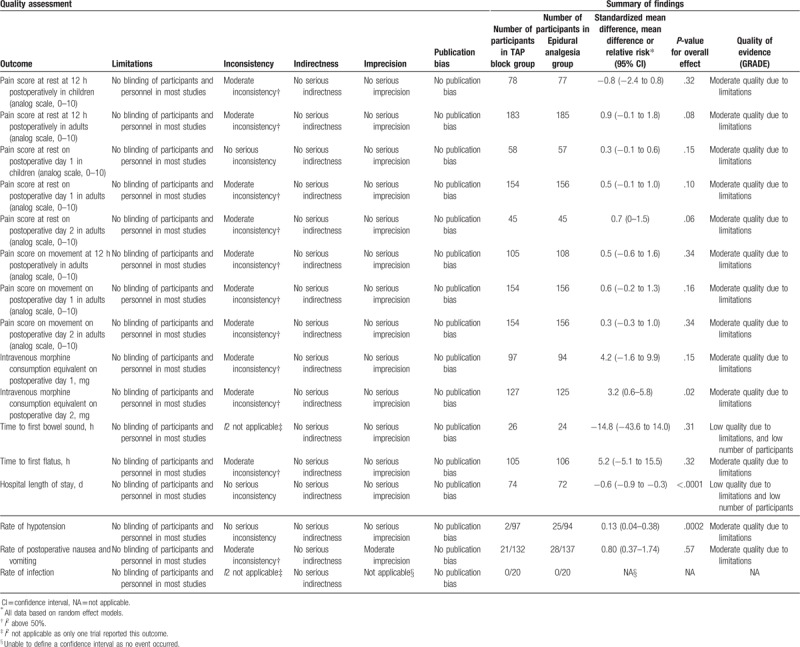
Summary of findings.

## 4 Discussion

This systematic review and meta-analysis compared the analgesic efficacy and side effects of TAP block versus epidural analgesia in pediatric and adult patients. Based on 10 randomized controlled trials, including a total of 505 patients, we demonstrated that both techniques provide equivalent analgesia after abdominal surgery, with a moderate level of evidence for our primary outcome, pain score at rest on postoperative day 1.

This finding is notable but decision making around postoperative analgesia after abdominal surgery is often driven by additional considerations such as a drive to enhance recovery of bowel function.^[25]^ Enhanced recovery after surgery care pathways emphasize analgesia toward achieving this goal including the use of epidural analgesia. Due to limited reporting of these outcomes, we were unable to draw any meaningful conclusions on the relative benefit of the 2 techniques for this goal.

The equivalent analgesic efficacy of both techniques should be properly balanced with consideration of the risk of hypotensive episodes associated with epidural analgesia and the reduced length of stay in patients receiving a TAP block demonstrated in this meta-analysis. While these outcomes may favor TAP block, it should be emphasized that neither technique is without drawbacks. The rate of failure or inadequate analgesia can be as high as 30% with either epidural analgesia,^[26,27]^ or TAP block.^[20]^ Finally, the rare but catastrophic risk of major complications associated with epidural analgesia such as epidural hematoma^[28]^ should be included in the considerations.

The results of our subgroup analysis suggest no difference between the subcostal and intercostoiliac approach when compared with epidural analgesia. This finding may not be surprising as the local anesthetic in both cases spreads transversally within a fascia plane to block the same targeted nerves. However, it should be highlighted that there is variation within the included studies for the surgical procedure location. The majority of trials employing a subcostal approach examined abdominal laparotomies with 2 trials of the intercostoiliac approach examining herniotomy. This heterogeneity may have accounted to some extent for the lack of difference seen between the subgroups.

There are additional notable limitations to this meta-analysis. First, as we describe above, we were unable to draw any robust conclusion regarding the impact of analgesic technique on the functional outcomes such as time to first bowel sounds. Consequently, the existing literature would benefit from additional trials employing a consistent methodology to better explore the relative functional impacts of TAP block compared to epidural analgesia. Second, although there were 7 different primary outcomes among the 10 included trials, we do not think that this myriad of different endpoints alters the validity of our results. We elected to define our primary one as pain score at rest on postoperative day 1, as we believe that it reflects the clinical comfort of the patients and therefore relevant for the daily practice of the anesthesiologist. Moreover, despite our attempt to group trials according to epidural and caudal analgesia, and despite the consistent regime of local anesthetics administered, the coefficient of heterogeneity remained elevated.

In conclusion, there is moderate evidence that TAP block and epidural analgesia are equally effective in treating postoperative pain in both pediatric and adult patients. Additional trials with robust methodology would better define the functional impact of each technique before supporting a stronger recommendation for TAP block, which is associated with fewer episodes of hypotension and reduced length of stay.

## Acknowledgments

The authors are grateful to Mrs Isabelle von Kaenel (Head librarian, Lausanne University Hospital, Lausanne, Switzerland) for the assistance in the literature search.

## Author contributions

Moira Baeriswyl: This author helped search the literature and prepare the primary manuscript.

Frank Zeiter: This author helped assess the articles and analyze the data.

Denis Piubellini: This author helped extract the data.

Kyle Robert Kirkham: This author helped assess the articles and edit the manuscript.

Eric Albrecht: This author helped design the study, search the literature, assess the articles, analyze the data, and write the manuscript.

**Conceptualization:** Eric Albrecht.

**Formal analysis:** Frank Zeiter.

**Methodology:** Moira Baeriswyl, Frank Zeiter, Denis Piubellini, Kyle Robert Kirkham, Eric Albrecht.

**Project administration:** Eric Albrecht.

**Supervision:** Eric Albrecht.

**Validation:** Eric Albrecht.

**Writing – original draft:** Moira Baeriswyl.

**Writing – review & editing:** Kyle Robert Kirkham, Eric Albrecht.
